# Clinical Characteristics and Immune Injury Mechanisms in 71 Patients with COVID-19

**DOI:** 10.1128/mSphere.00362-20

**Published:** 2020-07-15

**Authors:** Yingjie Wu, Xiaoxing Huang, Jiaxing Sun, Tian Xie, Yufei Lei, Jamal Muhammad, Xinran Li, Xingruo Zeng, Fuling Zhou, Hong Qin, Liang Shao, Qiuping Zhang

**Affiliations:** a Department of Pathology, Zhongnan Hospital of Wuhan University, Wuhan, People’s Republic of China; b Department of Blood Transfusion, Zhongnan Hospital of Wuhan University, Wuhan, People’s Republic of China; c Department of Immunology, School of Basic Medical Science, Wuhan University, Wuhan, People’s Republic of China; d School of Biomedical Sciences, Li Ka Shing Faculty of Medicine, The University of Hong Kong, Hong Kong, China; e Department of Medical Insurance Management, Zhongnan Hospital of Wuhan University, Wuhan, China; f Department of Hematology, Zhongnan Hospital of Wuhan University, Wuhan, China; National Institute of Allergy and Infectious Diseases

**Keywords:** coronavirus, SARS-CoV-2, COVID-19, immunological characteristics, inflammatory cytokine storm, infection

## Abstract

The outbreak of coronavirus disease 2019 (COVID-19), caused by the novel coronavirus severe acute respiratory syndrome coronavirus 2 (SARS-CoV-2), has caused a threat to global health. The mortality rate of severely ill patients in the early stage is 32.5%. The exacerbation of the condition and death of patients are closely associated with inflammatory cytokine storms, which are caused by excessive activation of the immune and complement systems as well as the coinfection of other pathogens. However, the immunological characteristics and the mechanisms underlying inflammatory storms have not been well elucidated. Here, we analyzed the clinical and immunological characteristics of 71 confirmed COVID-19 patients. Based on the National Health Commission of China (NHCC) guidelines, patients were stratified into mild and severe types. We compared the clinical and laboratory data obtained from electronic medical records between the two types. In regard to the hematological parameters, COVID-19 patients showed decreased erythrocyte count, hemoglobin, hematocrit, lymphocyte count, eosinophil count, and complement C1q, whereas neutrophils, C-reactive protein, and procalcitonin were significantly increased, especially in severe cases. We also found that CD3^+^ CD4^+^ T lymphocytes, CD3^+^ CD8^+^ T lymphocytes, CD19^+^ B lymphocytes, and CD16^+^ CD56^+^ NK cells in the peripheral blood of all patients were decreased. In addition, CD3^+^ CD8^+^ T lymphocytes, CD16^+^ CD56^+^ NK cells, and complement C1q in severely ill patients decreased more significantly. Additionally, interleukin 6 (IL-6) elevation was particularly prominent in all patients, especially in severe cases. These results suggest that CD3^+^ CD8^+^ T lymphocytes, CD16^+^ CD56^+^ NK cells, C1q as well as IL-6 may play critical roles in the inflammatory cytokine storm. The dysregulation of these aforementioned immune parameters, along with bacterial coinfection, were the important causes of exacerbation of the patients’ condition and death. This study improves our understanding of the immune dysregulation of COVID-19 and provides potential immunotherapeutic strategies.

**IMPORTANCE** The dysregulation of CD3^+^ CD8^+^ T lymphocytes, CD16^+^ CD56^+^ NK cells, C1q as well as IL-6, along with bacterial coinfection, were important causes of exacerbation of the patients’ condition and death.

## Abstract (precis)

The dysregulation of CD3^+^ CD8^+^ T lymphocytes, CD16^+^ CD56^+^ NK cells, C1q as well as IL-6, along with bacterial coinfection, were important causes of exacerbation of the patients’ condition and death.

## INTRODUCTION

The outbreak of coronavirus disease 2019 (COVID-19), caused by a novel coronavirus (severe acute respiratory syndrome coronavirus 2 [SARS-CoV-2]), has posed a threat to public health worldwide (1, 2). What is more, COVID-19 was declared to be a global pandemic by the World Health Organization (WHO) on 11 March 2020 due to its rapid spread (3).

By 22 June 2020, more than 85,056 confirmed cases and 4,646 deaths were reported in China, and 9,034,363 confirmed cases and 466,789 deaths were reported in countries and regions outside China. An epidemiological study has reported that the fatality rate in severe cases at the early stage of this epidemic is 32.5% (1).

COVID-19 is usually characterized by influenza-like symptoms such as fever, cough, shortness of breath, and myalgia. Chest computerized tomography (CT) scans usually show bilateral pneumonia or ground-glass opacity in the lungs (4). Severe or critically ill cases present as severe complications, such as acute respiratory distress syndrome (ARDS), septic shock acute cardiac injury (ACI), and acute kidney injury (AKI) (5). Unfortunately, up to date, there are no confirmed effective drugs specific for the treatment of COVID-19 (6). Clinical observations have shown that the exacerbation of disease and death of COVID-19 patients were closely related to the inflammatory storm caused by the excessive activation of the immune system and combined other pathogen infections (7, 8). Recently, a report of a fatal case showed overactivation of T cells, increased CCR4^+^ CCR6^+^ Th17 and high cytotoxicity of CD8^+^ T cells in the peripheral blood, suggesting that severe immune-related injury might occur in severe COVID-19 patients (9). However, the immunological characteristics of COVID-19 patients and the mechanism underlying the inflammatory storms have not been fully clarified. In the present study, we enrolled 71 confirmed hospitalized COVID-19 patients and investigated their immune status. Our study might improve the understanding of immune dysregulation of COVID-19 and provide new insights for immunotherapy.

## RESULTS

### Epidemiological and clinical characteristics of 71 COVID-19 patients.

The basic epidemiological and clinical characteristics of 71 COVID-19 patients were summarized in Table 1. The median age of all patients was 61 years (interquartile range [IQR], 49 to 69 years). The median age of patients with severe cases was 62 years (IQR, 54 to 72 years), which was older than that of the patients with mild cases with the median age of 56 years (IQR, 38 to 66 years). However, the difference did not reach statistical significance. The proportion of males was higher than females in the mild and severe groups. In detail, there were 14 females (43.8%) and 18 males (56.2%) in the mild group, whereas there were 12 females (30.8%) and 27 males (69.2%) in the severe group. A majority of patients had fever, especially those with severe cases, who all had fever. Additionally, more than half of patients had fatigue (51 [71.8%]), dry cough (39 [54.9%]), and anorexia (43 [60.6%]). Of note, there were statistically significant differences in fatigue and dry cough between mild and severe cases (*P* < 0.05). All severe cases presented dyspnea, whereas only 12.5% of mild cases did (*P* < 0.001). In regard to the comorbidities, 16 patients (22.5%) had hypertension, 14 (19.7%) had diabetes, 12 (16.9%) had cardiovascular disease, 2 (2.8%) had chronic obstructive pulmonary disease, and 6 (8.5%) had malignancies. However, there were no statistically significant differences in the prevalence of these comorbidities between mild and severe cases.

**TABLE 1:** Baseline characteristics of 71 patients with COVID-19^*a*^

Characteristic	No. (%) of individuals with characteristic unless otherwise noted			*P* value
	Total (*n* = 71)	Mild (*n* = 32)	Severe (*n* = 39)	
Age (yr), median (IQR)	61 (49–69)	56 (38–66)	62 (54–72)	0.0892
Gender				0.2586
Female	26 (36.7)	14 (43.8)	12 (30.8)	
Male	45 (63.3)	18 (56.2)	27 (69.2)	
Clinical symptoms				
Fever	69 (95.8)	30 (93.8)	39 (100)	0.1133
Fatigue	51 (71.8)	19 (59.4)	32 (82.1)	0.0346
Dry cough	39 (54.9)	13 (40.6)	26 (66.7)	0.0282
Anorexia	43 (60.6)	19 (59.4)	24 (61.5)	0.8528
Myalgia	24 (33.8)	12 (37.5)	12 (30.8)	0.5508
Dyspnea	43 (60.6)	4 (12.5)	39 (100)	<0.001
Comorbidities				
Hypertension	16 (22.5)	4 (12.5)	12 (30.8)	0.0668
Diabetes	14 (19.7)	6 (18.8)	8 (20.5)	0.8526
Cardiovascular disease	12 (16.9)	3 (9.4)	9 (23.4)	0.1253
COPD	2 (2.8)	1 (3.1)	1 (2.6)	0.8870
Malignancy	6 (8.5)	1 (3.1)	5 (12.8)	0.1439

a Abbreviations: IQR, interquartile range; COPD, chronic obstructive pulmonary disease.

### Hematological and inflammatory parameters of COVID-19 patients.

**FIG 1:**
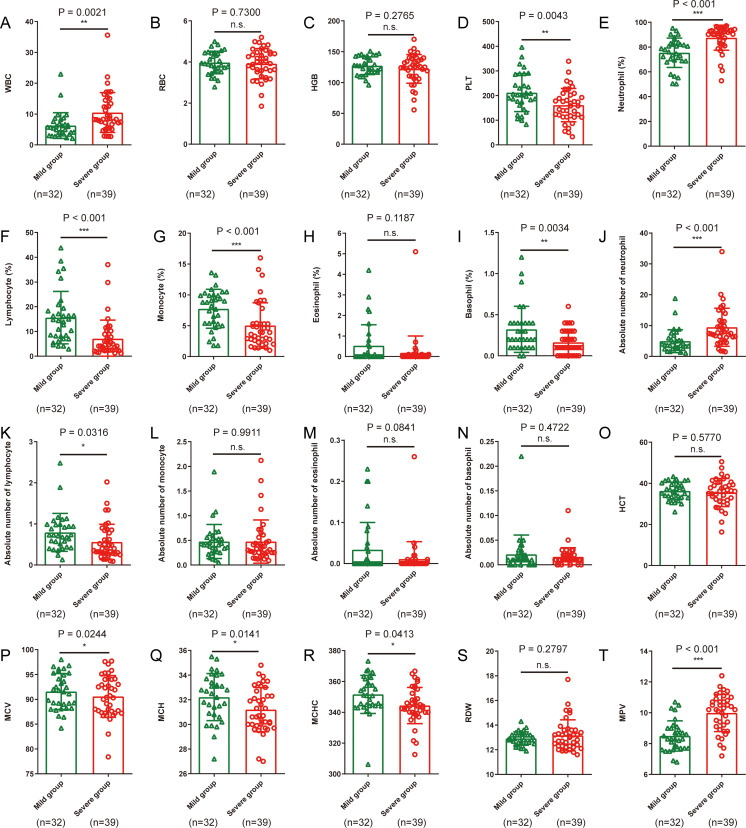
Differences in routine blood analysis characteristics between 32 patients with mild cases of COVID-19 and 39 patients with severe cases of COVID-19. (A to D) WBC, RBC, HGB, and PLT; (E to I) Percentages of neutrophil, lymphocyte, monocyte, eosinophils, and basophils; (J to N) Absolute numbers of neutrophils, lymphocytes, monocytes, eosinophils, and basophils; (O to T) HCT, MCV, MCH, MCHC, RDW. and MPV. Statistical significance is indicated as follows: *, *P* < 0.05; **, *P* < 0.01; ***, *P* < 0.001; n.s., not significant.

**FIG 2:**
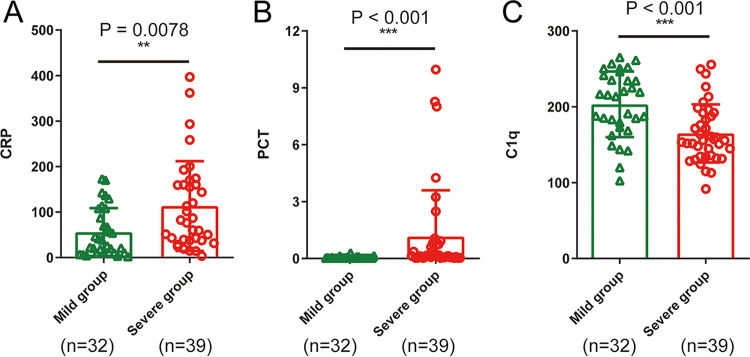
Differences in inflammation indicators between 32 mild and 39 severe COVID-19 patients. (A) CRP, (B) PCT and (C) C1q. **, *P* < 0.01; ***, *P* < 0.001.

As shown in Table 2 and Fig. 1, hematological and inflammatory parameters in COVID-19 patients were analyzed. Red blood cell (RBC), hemoglobin (HGB), lymphocyte percentage (LYMPH%), eosinophil percentage (EO%), lymphocyte count (LYMPH), eosinophil count (EO), and hematocrit (HCT) were reduced, whereas neutrophil percentage (NEUT%) and neutrophil count (NEUT) were elevated in COVID-19 patients. Compared with mild cases, severe cases showed higher white blood cell (WBC) count (*P* = 0.0021; Fig. 1A), NEUT% (*P* < 0.001; Fig. 1E), mean platelet volume (MPV) (*P* < 0.001; Fig. 1T), C-reactive protein (CRP) (*P* = 0.0078; Fig. 2A), procalcitonin (PCT) (*P* < 0.001; Fig. 2B) and lower platelets (PLT) count (*P* = 0.0043; Fig. 1D), LYMPH% (*P* < 0.001; Fig. 1F), LYMPH (*P* = 0.0316; Fig. 1K), and complement C1q (*P* < 0.001; Fig. 2C).

**TABLE 2:** Hematological parameters and inflammatory profiles of 71 patients with COVID-19 on hospital admission^*a*^

Hematological parameter or inflammatory profile	Normal range	Median (IQR)			*P* value
		Total (*n* = 71)	Mild (*n* = 32)	Severe (*n* = 39)	
Hematological parameters					
White blood cell (×10^9^/liter)	3.50 to 9.50	7.18 (4.64–9.77)	5.56 (3.54–7.41)	8.31 (6.66–14.46)	0.0021
Red blood cell (×10^12^/liter)	4.30–5.80	3.92 (3.56–4.45)	3.91 (3.56–4.45)	4.02 (3.52–4.47)	0.7300
Hemoglobin (g/liter)	130.00–175.00	126.00 (112.90–140.00)	127.40 (117.33–14.48)	125.00 (110.00–139)	0.2765
Platelets (×10^9^/liter)	125.00–350.00	181.00 (123.00–227.00)	203.00 (164.25–275.00)	158.00 (113.00–209.00)	0.0043
Neutrophil percentage (%)	40.0–75.0	83.8 (73.6–92.5)	77.1 (69.1–82.8)	91.1 (82.9–93.8)	<0.001
Lymphocyte percentage (%)	20.0–50.0	8.1 (4.1–15.1)	14.6 (8.1–19.1)	4.5 (2.4–8.9)	<0.001
Monocyte percentage (%)	3.0–10.0	5.3 (3.0–9.1)	8.8 (5.4–10.0)	3.9 (2.6–5.4）	<0.001
Eosinophil percentage (%)	0.4–8.0	0.0 (0.0–0.1)	0.0 (0.0–0.6)	0.0 (0.0–0.0)	0.1187
Basophils percentage (%)	0.0–1.0	0.2 (0.1–0.3)	0.2 (0.1–0.4)	0.1 (0.1–0.3)	0.0034
Neutrophil (×10^9^/liter)	1.80–6.30	6.30 (3.47–9.25)	3.65 (2.58–5.80)	7.66 (6.16–11.6)	<0.001
Lymphocyte count (×10^9^/liter)	1.10–3.20	0.57 (0.32–0.92)	0.72 (0.46–0.99)	0.40 (0.28–0.84)	0.0316
Monocyte count (×10^9^/liter)	0.10–0.60	0.38 (0.25–0.56)	0.41 (0.27–0.56)	0.33 (0.24–0.56)	0.9911
Eosinophil count (×10^9^/liter)	0.02–0.50	0.00 (0.00–0.01)	0.00 (0.00–0.38)	0.00 (0.00–0.00)	0.0841
Basophil count (×10^9^/liter)	0.00–0.06	0.01 (0.01–0.02)	0.01 (0.00–0.02）	0.01 (0.01–0.02）	0.4722
Hematocrit (%)	40.0–50.0	36.2 (32.2–40.2)	36.5 (33.4–40.0)	36.2 (31.4–40.4)	0.5770
MCV (fl)	82.00–100.00	91.10 (88.00–94.10)	91.65 (88.40–95.20)	90.40 (87.30–94.00)	0.0244
MCH (pg)	27.00–34.00	31.70 (30.10–33.10)	32.60 (30.83–33.63)	30.70 (30.00–32.70)	0.0141
MCHC (g/liter)	316.00–354.00	345.90 (341.20–357.00)	351.00 (344.30–361.08)	344.00 (339.20–352.30)	0.0413
RDW (%)	10.1–16.0	12.9 (12.4–13.4)	8.3 (7.7–9.0)	13.0 (12.3–13.6)	0.2797
Mean platelet volume (fl)	6.00–12.00	9.10 (8.30–10.50)	8.40 (7.70–9.13)	10.20 (9.05–10.83)	<0.001
Inflammatory profile					
C-reactive protein (mg/liter)	0.00–10.00	55.60 (20.93–131.45)	40.45 (10.30–102.90)	79.44 (37.89–160.00)	0.0078
Procalcitonin (ng/ml)	<0.05	0.06 (0.03–0.19)	0.03 (0.03–0.05)	0.16 (0.06–0.79)	<0.001
Complement C1q (mg/liter)	159.00–233.00	179.30 (148.10–219.50)	214.75 (174.73–240.03)	155.70 (134.60–191.80)	<0.001

a Abbreviations: IQR, interquartile range; MCV, mean red blood cell volume; MCH, mean hemoglobin content; MCHC, mean hemoglobin concentration; RDW, red blood cell distribution width.

As shown in Table 2 and Fig. 2, analysis of inflammatory parameters in COVID-19 patients showed that, compared with mild cases, severe cases gained higher C-reactive protein (CRP) (*P* = 0.0078), procalcitonin (PCT) (*P* < 0.001), and lower complement C1q (*P* < 0.001).

### Lymphocyte profile in COVID-19 patients.

**FIG 3:**
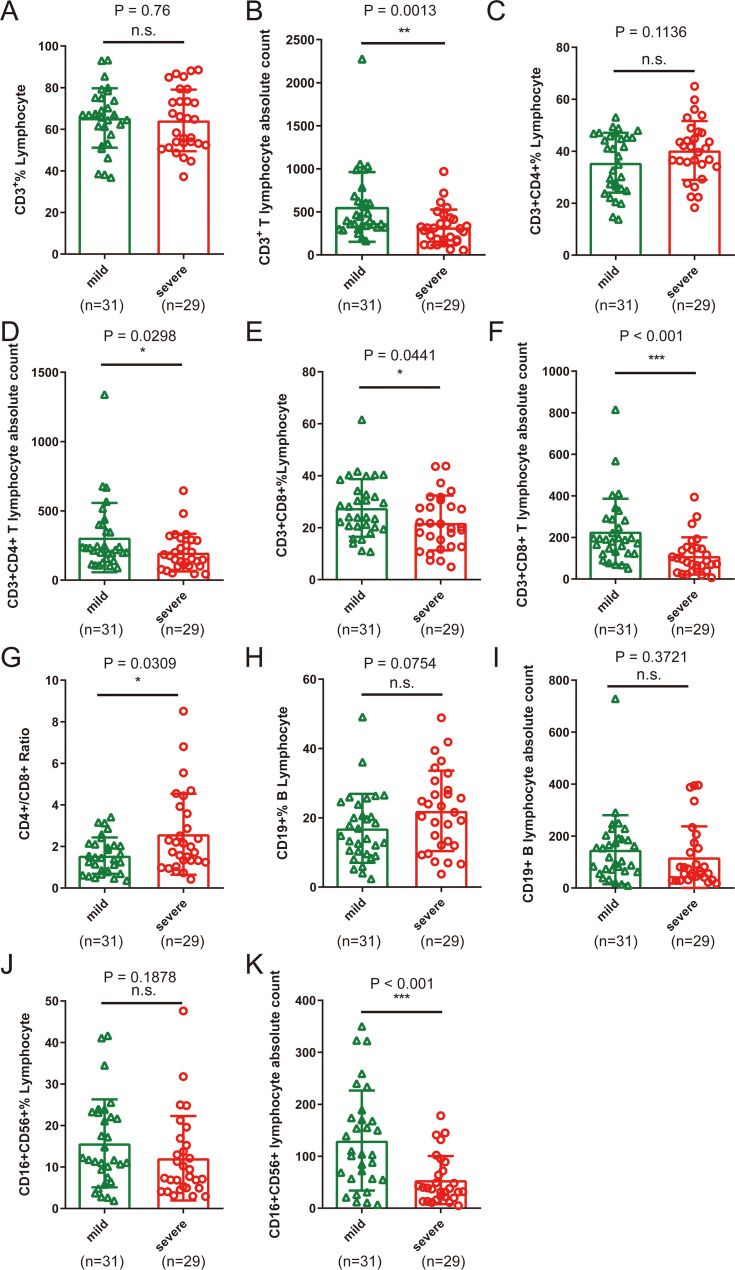
Peripheral blood lymphocyte subset characteristics between 31 mild and 29 severe COVID-19 patients. (A, C, E, H, and J) Percentages of CD3^+^ lymphocytes, CD3^+^ CD4^+^ lymphocytes, CD3^+^ CD8^+^ lymphocytes, CD19^+^ lymphocytes, and CD16^+^ CD56^+^ lymphocytes; (B, D, F, I, and K) CD3^+^ Abs Cnt, CD3^+^ CD4^+^ Abs Cnt, CD3^+^ CD8^+^ Abs Cnt, CD19^+^ Abs Cnt, and CD16^+^ CD56^+^ Abs Cnt; (G) CD4^+^ /CD8^+^ ratio. *, *P* < 0.05; **, *P* < 0.01; ***, *P* < 0.001; n.s. not significant.

As shown in Table 3 and Fig. 3, in all patients, CD3^+^ CD4^+^ T lymphocyte absolute count (CD3^+^ Abs Cnt), CD3^+^ CD4^+^ T lymphocyte absolute count (CD3^+^ CD4^+^ Abs Cnt), CD3^+^ CD8^+^ T lymphocyte absolute count (CD3^+^ CD8^+^ Abs Cnt), CD19^+^ B lymphocyte absolute count (CD19^+^ Abs Cnt), and CD16^+^ CD56^+^ lymphocyte absolute count (CD16^+^ CD56^+^ Abs Cnt) were obviously decreased, whereas CD3^+^ CD4^+^ T lymphocyte percentage (CD3^+^ CD4^+^% Lym), CD3^+^ CD8^+^ T lymphocyte percentage (CD3^+^ CD8^+^% Lym), and CD19^+^ B lymphocyte percentage (CD19^+^% Lym) were significantly elevated.

**TABLE 3:** Peripheral blood lymphocyte subsets of 60 patients with COVID-19 on hospital admission^*a*^

Peripheral blood lymphocyte subset or parameter	Normal range	Median (IQR)			*P* value
		Total (*n* = 60)	Mild (*n* = 31)	Severe (*n* = 29)	
CD3^+^% Lym	38.56–70.06	65.92 (53.31–74.74)	66.85 (57.53–75.05)	64.75 (52.75–76.36)	0.76
CD3^+^ Abs Cnt	805–4459	359 (256–558)	399 (324–626)	306 (167–422)	0.0013
CD3^+^ CD4^+^% Lym	14.21–36.99	40.32 (27.66–46.56)	38.13 (25.70–45.80)	41.09 (34.51–47.30)	0.1136
CD3^+^ CD4^+^ Abs Cnt	345–2350	201 (128–315)	234 (156–401)	153 (102–289)	0.0298
CD3^+^ CD8^+^% Lym	13.24–38.53	22.86 (17.05–31.83)	24.10 (20.25–34.23)	20.19 (12.69–29.36)	0.0441
CD3^+^ CD8^+^ Abs Cnt	345–2350	143 (72–198)	191 (125–288)	88 (45–147)	<0.001
CD4^+^/CD8^+^ ratio	0.96–2.05	1.61 (0.99–2.49)	1.46 (0.78–2.11)	1.99 (1.28–3.75)	0.0309
CD19^+^% Lym	10.86–28.03	18.62 (10.42–25.92)	14.88 (9.6023.87)	21.59 (12.03–29.31)	0.0754
CD19^+^ Abs Cnt	240–1317	84 (47–188)	119 (66–192)	65 (36–164)	0.3721
CD16^+^ CD56^+^% Lym	7.92–33.99	11.20 (5.97–21.18)	11.77 (7.53–23.12)	7.71 (4.95–16.07)	0.1878
CD16^+^ CD56^+^ Abs Cnt	210–1514	67 (31–144)	104 (57–174)	38 (21–81)	<0.001

a Abbreviations: IQR, interquartile range; Lym, lymphocytes; Abs Cnt, absolute count; %, percentage.

In addition, the CD16^+^ CD56^+^ NK cell percentage (CD16^+^ CD56^+^% Lym) was significantly reduced in the severe group. Severe patients showed lower CD3^+^ Abs Cnt (*P* = 0.0013; Fig. 3B), CD3^+^ CD4^+^ Abs Cnt (*P* = 0.0298; Fig. 3D), CD3^+^ CD8^+^ Abs Cnt (*P* < 0.001; Fig. 3F), as well as CD16^+^ CD56^+^ Abs Cnt (*P* < 0.001; Fig. 3K), and a higher CD4^+^/CD8^+^ ratio (*P* = 0.0309; Fig. 3G) than that of mild ones.

### Immune cytokines in COVID-19 patients.

**FIG 4:**
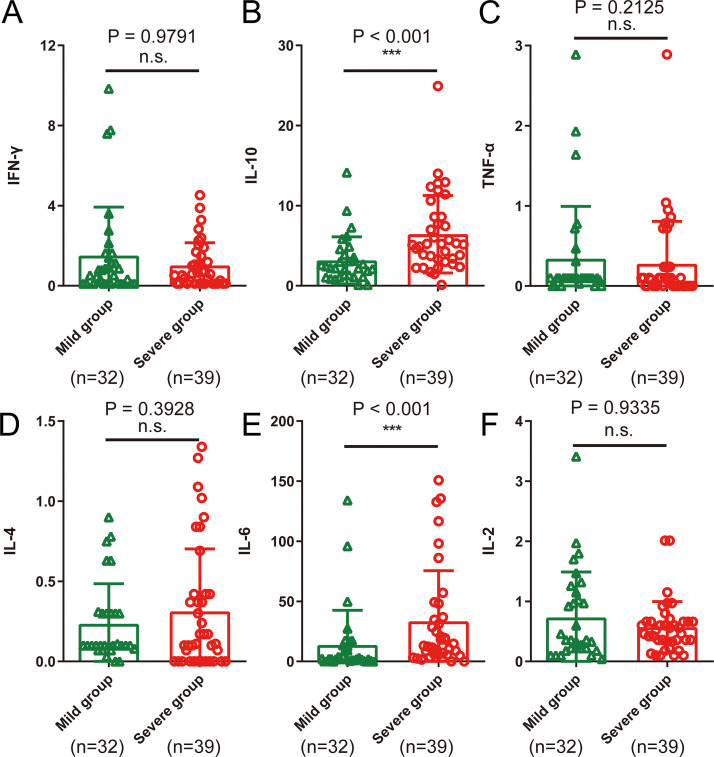
Serum cytokine characteristics between 32 mild and 39 severe COVID-19 patients. (A) IFN-γ; (B) IL-10; (C) TNF-α; (D) IL-4; (E) IL-6; (F) IL-2. *, *P* < 0.05; **, *P* < 0.01; ***, *P* < 0.001.

As shown in Table 4 and Fig. 4, interleukin 6 (IL-6) was elevated in total COVID-19 patients as well as in the severe group, while IL-10 was increased only in severe cases. In addition, the IL-6 (*P* < 0.001; Fig. 4E) and IL-10 (*P* < 0.001; Fig. 4B) levels in the severe group were higher than those in the mild group. Furthermore, the IL-2 level was higher and IL-4 level was lower in severe cases than that of mild ones, but the difference was not significant. However, tumor necrosis factor alpha (TNF-α) and gamma interferon (IFN-γ) showed no significant difference between the two groups.

**TABLE 4:** Serum cytokines of 71 patients with COVID-19 on hospital admission

Cytokine	Value for cytokine				*P* value
	Normal range	Total (*n* = 71)	Mild (*n* = 32)	Severe (*n* = 39)	
IFN-γ (pg/ml)	0.10–18.00	0.58 (0.11–1.63)	0.66 (0.11–1.57)	0.58 (0.11–1.69)	0.9791
IL-10 (pg/ml)	0.10–5.00	3.82 (2.17–7.27)	2.34 (1.16–4.41)	5.23 (3.31–10.64)	<0.001
TNF-α (pg/ml)	0.10–23.00	0.10 (0.00–0.24)	0.10 (0.10–0.26)	0.1 (0.00–0.24)	0.2125
IL-4 (pg/ml)	0.10–3.20	0.17 (0.07–0.42)	0.24 (0.10–0.48)	0.11 (0.00–0.42)	0.3928
IL-6 (pg/ml)	0.10–2.90	9.53 (2.11–28.71)	2.21 (0.83–13.22)	18.15 (5.91–49.24)	<0.001
IL-2 (pg/ml)	0.10–4.10	0.46 (0.22–0.92)	0.36 (0.22–1.23)	0.52 (0.33–0.66)	0.9335

## DISCUSSION

The recent and ongoing outbreak of COVID-19 has caused a widespread pandemic in the world. Emerging studies have demonstrated that immune damage and the development of inflammatory storms are critical to the deterioration of the condition of patients with coronavirus pneumonia, such as SARS and Middle East respiratory syndrome (MERS) (10–12). The exacerbation and death of COVID-19 patients appear to be related to inflammatory storms, caused by excessive activation of the immune system and combined bacterial infection, although the immunological characteristics of COVID-19 and the mechanisms underlying the inflammatory storms have not been fully elucidated. In our study, we systematically analyzed the clinical characteristics, routine blood indexes, inflammation and infection indicators, complement C1q, lymphocyte subsets, and cytokines in 71 confirmed COVID-19 patients and compared the immunological characteristics between the mild and severe cases.

Consistent with several published papers (13, 14), we observed that the clinical symptoms of COVID-19 included but were not limited to fever, cough, myalgia, diarrhea, and dyspnea. Some patients had underlying illnesses, including hypertension, diabetes, cardiovascular disease, chronic obstructive pulmonary disease, and malignancies.

Our analysis has shown that both mild and severe cases had normal levels of RBC, HGB, mean red blood cell volume (MCV), mean hemoglobin content (MCH), and mean hemoglobin concentration (MCHC). However, these parameters exhibited a tendency to decrease, especially in the severe patients. This phenomenon might be due to the poor appetite and abnormal diet after SARS-CoV-2 infection, resulting in the lack of synthetic materials for hemoglobin such as iron. Iron deficiency could influence the formation of red blood cells (RBCs), eventually causing anemia. In this regard, physicians should be cautious about the malnutrition of COVID-19 patients, especially the severely ill patients in intensive care units (ICUs). On the other hand, appropriate blood transfusion is recommended for patients with severe anemia. MPV was significantly higher in severe cases than in mild cases, but both were within the normal range. Platelets are produced by megakaryocytes and gradually decrease in size after formation. Therefore, we speculate that our findings may be due to the relatively low number of platelets in patients with severe disease, leading to increased platelet regeneration and compensation.

The increased peripheral neutrophil percentage and count may be due to the attack on the immune system in COVID-19 patients, resulting in decreased immunity leading to the coinfection of bacteria. In the present study, levels of peripheral white blood cells and neutrophils were higher in patients with severe cases than in patients with mild cases, which might be due to the higher incidence of bacterial infection and the other pathogens in severe cases. Moreover, PCT is an indicator of bacterial infection and is positively correlated with the severity of the disease. The low level of PCT (<0.05 ng/ml) hints that the patients did not have a coinfection of bacteria. Our study revealed that the level of PCT in severe cases was significantly increased, and there was a significant difference compared with the level in mild cases, indicating that severe patients had more severe bacterial infection. We also found that patients with pneumonia had significantly reduced peripheral blood eosinophil counts and percentages, which may drop to zero in either mild or severe cases. The reduction in peripheral blood eosinophils usually occurs in typhoid fever, which results from typhoid endotoxin-mediated inhibition of bone marrow proliferation. In the autopsy reports of SARS patients, the white pulp of the spleen was severely atrophied in multiple cases (15), and the ultramicropathological results of the SARS patient autopsies showed that SARS virus caused infection of multiple organs, indicating that the virus can invade cells in many tissues, especially the lungs and immune organs (16). Therefore, we speculate that the new coronavirus may affect bone marrow and other immune organs such as the spleen and lymph nodes, leading to the reduction in eosinophils and other immune cells. In particular, all COVID-19 patients had decreased peripheral blood lymphocyte counts and lymphocyte percentages. Compared with mild cases, the peripheral blood lymphocyte counts and percentages were even lower in severe cases, which might be due to the recruitment of lymphocytes from the peripheral blood to the tissues and organs by the SARS-CoV-2 virus.

By analyzing the lymphocyte profile, we found that the counts of CD3^+^ T lymphocytes, CD3^+^ CD4^+^ T lymphocytes, CD3^+^ CD8^+^ T lymphocytes, CD19^+^ B lymphocytes, and CD16^+^ CD56^+^ NK cells in the peripheral blood were obviously decreased. The percentage of these lymphocyte subsets was increased, except for CD16^+^ CD56^+^ NK cells. In particular, the percentage of CD16^+^ CD56^+^ NK cells in severe cases was lower. These results indicated that for all patients, peripheral blood T lymphocytes, B lymphocytes, and NK cells are downregulated, with NK cells even more prominently downregulated. Compared with mild COVID-19 patients, severe COVID-19 patients had lower peripheral CD3^+^ T lymphocyte count and CD3^+^ CD4^+^ T lymphocyte count, especially CD3^+^ CD8^+^ T lymphocytes and CD16^+^ CD56^+^ NK cells. The reduction in NK cells also occurs in SARS patients, which is the result of redistribution to target organs and a SARS virus-activated apoptosis mechanism (17). Therefore, we hypothesized that recruitment and apoptosis of cells would also induce the NK cell reduction in SARS-CoV-2-infected patients. In addition, the CD4^+^/CD8^+^ ratio in severe cases was higher than that in mild cases, which indicated that compared with CD3^+^ CD4^+^ T cells, CD3^+^ CD8^+^ T lymphocytes in patients with severe disease have a greater degree of lymphatic decline. These results indicate that various types of lymphocytes were decreased, and this reason may be that the cells recruited into local tissues or SARS-CoV-2 virus infection inhibited bone marrow function. In addition, dysregulation of CD3^+^ CD8^+^ T lymphocytes and NK cells may play more important roles in the worsening of the disease, but the mechanism was still unclear. We agreed with the perspective of Vardhana and Wolchok (1) who hypothesized that CD8^+^ effector T cells might be redistributed and be consumed during the battle for SARS-CoV-2 in human bodies, especially in the severe cases of COVID-19. As we know, effective viral clearance needs both CD8^+^ effector T cell-mediated effective killing of virally infected cells and CD4^+^ T cell-dependent enhancement of CD8^+^ T and B cell responses. Following viral clearance, these virus-specific T cells finally undergo cell apoptosis. Some studies have reported that hyperactivation of CCR4^+^ CCR6^+^ Th17 and high cytotoxicity of CD8^+^ T lymphocytes existed in patients with pneumonia (9).

Previous studies have reported that serum levels of the proinflammatory factors IL-1β, IL-6, IL-12, IFN-γ, interferon-inducible protein of 10 kDa (IP-10), and monocyte chemotactic peptide 1 (MCP-1) were closely related to increased lung injury in SARS patients (10). Furthermore, another study found that the upregulation of IFN-γ, TNF-α, IL-15, and IL-17 in serum was closely related to the poor prognosis of MERS patients (11). Although cytokine profiles in the peripheral blood of patients with COVID-19 have been reported (below), there is still some controversy and uncertainty about the expression levels of cytokines in the progression of COVID-19. Huang et al. found that, compared with non-ICU patients, IL-2, IL-10, and TNF-α levels in the peripheral blood of ICU patients were significantly increased, whereas IL-6, IL-4, and IFN-γ were not significantly different (14). Chen et al. found that the IL-6 level in the peripheral blood of patients with COVID-19 was higher than that of healthy patients, and there was no significant difference in IL-10 or TNF-α levels between patients with normal, severe, and critical disease (18). Our results showed significant increases in IL-6 and IL-10 in the peripheral blood of patients with severe cases of COVID-19 compared to the levels in patients with mild cases. TNF-α, IFN-γ, and IL-2 were also higher while IL-4 was lower in severe COVID-19 cases than in mild cases, but the difference was not significant. In summary, in combination with our results and prior reports, IL-6 may play a key role in triggering inflammatory storms in COVID-19 patients. IL-10, a negative regulator of immunity, is increased in this process, which may be caused by the body’s immune protection mechanism. However, the specific characteristics and functions of the local immune microenvironment of IL-6 and IL-10 in damaged organs need to be further explored. In addition to the previously reported method of tocilizumab targeting the IL-6 receptor, there are other clinical methods to target the IL-6/IL-6 receptor (IL-6R) pathway, such as siltuximab (targeting IL-6, approved in Castleman disease) and sarilumab (targeting IL-6R, approved in rheumatoid arthritis), which may also act as potential drugs for the treatment of COVID-19 (2, 7, 12).

In addition, CRP is an indicator of the body’s inflammatory response after pathogen infection. In our study, the CRP level in the blood of patients with COVID-19 was significantly increased, while there was a significant difference between patients with severe and mild cases. Complement C1q levels in severe cases were lower than the levels in mild cases, indicating that the body activated the complement system and consumed C1q molecules after SARS-CoV-2 virus infection. On the one hand, the activation of the complement system plays an anti-infection role, and on the other hand, a large number of inflammatory mediators, including anaphylatoxin and chemokines, can be produced during the activation process, which further aggravates the inflammatory factor storm and promotes the damage of target organs. Therefore, transfusing fresh plasma from recovered COVID-19 patients to treat critically ill COVID-19 patients may exhibit a potential curative effect. The mechanisms may involve the following two aspects: specific antibodies may play a role in the resistance against viral infection, and other components in the plasma may also play an important role in correcting the disorder of immune function and the recovery of the damaged hematopoietic system.

In conclusion, our study demonstrated characteristics of hematological indexes, inflammatory parameters, various immune cells, and cytokines in 71 patients with COVID-19. The results suggest that CD3^+^ CD8^+^ T lymphocytes, CD16^+^ CD56^+^ NK cells, C1q, and IL-6 may play important roles in the inflammatory cytokine storm and, with bacterial coinfection, were the important causes for patients' exacerbation and death. On the basis of this finding, our results might shed new light on understanding the immune dysregulation and immunotherapy of COVID-19 patients.

## MATERIALS AND METHODS

### Patient information and data collection.

Seventy-one confirmed COVID-19 patients hospitalized in Zhongnan Hospital of Wuhan University during the period from 29 December 2019 to 20 February 2020 were included in this study. This study was approved by the Ethics Committee of Zhongnan Hospital of Wuhan University. All patients met the criteria for clinical diagnosis according to the National Health Commission of China (NHCC) guidelines (19) on COVID-19 for diagnosis and disease severity triage. Clinical and laboratory information such as age, sex, medical history, comorbidities, signs and symptoms, severity assessment on admission, and laboratory examinations were obtained from electronic medical records. On hospital admission, the common (mild) cases were those who had only fever, respiratory symptoms, and pneumonia according to chest radiography. Severe cases needed to meet one of the following criteria: (i) respiratory distress (respiratory rate [RR] of ≥30/min), (ii) resting blood oxygen saturation ≤ 93%, or (iii) arterial blood oxygen partial pressure (PaO2)/FiO2 ≤ 300 mm Hg. Critically ill cases needed to meet one of the following conditions: (i) respiratory failure and mechanical oxygenation requirement, (ii) shock, and (iii) development of organ failure requiring intensive care unit (ICU) care.

### Real-time reverse transcription-PCR assay.

Real-time reverse transcription-PCR (real-time RT-PCR) assessment of laryngeal swab samples was performed as reported previously (13). The total RNA of the virus was extracted within 2 h with an RNA isolation kit (Zhongzhi, Wuhan, China) according to the product manual. Two targeted genes, open reading frame 1ab (*ORF1ab*) and nucleocapsid protein (N), were amplified and tested at the same time. Target 1 (*ORF1ab*) was as follows: forward primer CCCTGTGGGTTTTACACTTAA and reverse primer ACGATTGTGCATCAGCTGA, and the probe was 5′-VIC-CCGTCTGCGGTATGTGGAAAGGTTATGG-BHQ1-3′ (BHQ stands for black hole quencher). Target 2 (N) was as follows: forward primer GGGGAACTTCTCCTGCTAGAAT and reverse primer CAGACATTTTGCTCTCAAGCTG, and the probe was 5′-FAM-TTGCTGCTGCTTGACAGATT-TAMRA-3′ (FAM stands for 6-carboxyfluorescein, and TAMRA stands for 6-carboxytetramethylrhodamine). The real-time RT-PCR assay was performed with a SARS-CoV-2 nucleic acid detection kit (Shanghai Biogerm Medical Technology Co., Ltd.) according to the manufacturer’s protocol. A cycle threshold (*C_T_*) value of less than 37 was defined as a positive test result, and a *C_T_* value greater than 40 was defined as a negative test. When the *C_T_* value was between 37 and 40, the samples were subjected to retesting for further confirmation.

### Lymphocyte subset analysis.

The lymphocyte test kit (BD Multitest 6-color TBNK reagent; BD Biosciences, San Jose, CA, USA) was used for lymphocyte subset analysis. The test was performed according to the product manual. Anticoagulated whole blood (50 μl) and BD Multitest 6-color TBNK reagent (20 μl) were gently mixed and incubated for 15 min in the dark at room temperature. Then, 1× BD fluorescence-activated cell sorting (FACS) lysing solution (450 μl) was added to the tube. The tube was covered and vortexed gently to mix. After incubating in the dark at room temperature for 15 min, the samples were analyzed on a flow cytometer. The fluorescence signals were measured by a BD FACSCanto II flow cytometer (BD Biosciences, San Jose, CA, USA).

### Plasma cytokine analysis.

Plasma cytokines (IL-2, IL-4, IL-6, IL-10, TNF-α, and IFN-γ) were detected with a human Th1/Th2 subset detection kit (flow fluorescence method) (Hangzhou Cellgene Biotech Co., Ltd, Hangzhou, China). Briefly, the cytokines were measured by multiple-color flow cytometry with human monoclonal anti-IL-2 labeled with phycoerythrin (anti-IL-2-PE), anti-IL-4-PE, anti-IL-6-PE, anti-TNF-α-PE, and anti-IFN-γ-PE antibodies according to the manufacturer’s instructions. Plasma (25 μl) from each patient was drawn into a tube, and then 25 μl fluorescence detection reagent was added to the tube. The tube was covered, vortexed gently to mix the contents of the tube, and incubated in the dark at room temperature for 2.5 h. Then, we added 1 ml of 1× phosphate-buffered saline (PBS). After centrifugation (5 min, 200 × *g*, at room temperature), the sediment was suspended in 100 μl PBS. The samples were analyzed on a flow cytometer. The fluorescence signals were measured by a BD FACSCanto II flow cytometer (BD Biosciences, San Jose, CA, USA).

### Statistical analysis.

Categorical variables are described by frequency and percentage, and continuous variables are described by median and interquartile range (IQR) values. When the data were normally distributed, the independent sample *t* test was used to compare the mean values of continuous variables; otherwise, the Mann-Whitney U test was used. Differences in categorical variables were assessed using the χ^2^ test, and Fisher’s exact test was used in the case of limited data. All statistical analyses were performed using GraphPad Prism (GraphPad Company, San Diego, CA, USA) version 6.0 software. A *P* value of <0.05 was considered statistically significant.
